# Supplementary material for: K+-Driven Cl−/HCO3− Exchange Mediated by Slc4a8 and Slc4a10
Source: Int J Mol Sci. 2024 Apr 22;25(8):4575. doi: 10.3390/ijms25084575 (PMC11050268; doi:10.3390/ijms25084575)
Supplement: Supplementary file 1 [file ijms-25-04575-s001.zip › ijms-2944687-supplementary.pdf]

Supplementary Figure 1

```
              (…)
mSlc4a10    RISAIESLFGASMTGIAYSLFGGQPLTILGSTGPVLVFEKILFKFCKEYGLSYLSLRASI     597
rSlc4a8     RISAIESLFGASMTGIAYSLFAGQPLTILGSTGPVLVFEKILFKFCKDYALSYLSLRACI     566
mSlc4a8     RISAIESLFGASMTGIAYSLFAGQPLTILGSTGPVLVFEKILFKFCKDYALSYLSLRALI     564
            *********************.************************:*.******** *

mSlc4a10    GLWTATLCIILVATDASSLVCYITRFTEEAFASLICIIFIYEALEKLFELSETYPINMHN     657
rSlc4a8     GLWTAFLCIVLVATDASSLVCYITRFTEEAFASLICIIFIYEAIEKLIHLAETYPIHMHS     626
mSlc4a8     GLWTAFLCIVLVATDASSLVCYITRFTEEAFASLICIIFIYEAIEKLIHLAETYPIHMHS     624
            ***** ***:*********************************:***:.*:*****:**.
            (…)
mSlc4a10    AIGIPSPKLQVPSVFKPTRDDRGWFVTPLGPNPWWTIIAAIIPALLCTILIFMD831QQIT835AV   837
rSlc4a8     LIGVPSPKLQVPSVFKPTRDDRGWFISPIGPNPWWTVIAAIIPALLCTILIFMD800QQIT804AV   806
mSlc4a8     LIGVPSPKLQVPNVFKPTRDDRGWFINPIGPNPWWTVIAAIIPALLCTILIFMD798QQIT802AV   804
            **:********.************:.*:*******:****************   ****  **

mSlc4a10    IINRKEHKLKKGCGYHLDLLMVAVMLGVCSIMGLPWFVAAT878VLSITHVNSLKLESECSAP     897
rSlc4a8     IINRKEHKLKKGCGYHLDLLVVAIMLGVCSLMGLPWFVAAT847VLSITHVNSLKLESECSAP     866
mSlc4a8     IINRKEHKLKKGCGYHLDLLMVAVMLGVCSIMGLPWFVAAT845VLSITHVNSLKLESECSAP     864
            ********************:**:******:**********   *******************

mSlc4a10    GEQPKFLGIREQRVTGLMIFILMGSSVFMTSILKFIPMPVLYGVFLYMGASSLKGIQLFD     957
rSlc4a8     GEQPKFLGIREQRVTGLMIFVLMGCSVFMTAVLKFIPMPVLYGVFLYMGVSSLQGIQFFD     926
mSlc4a8     GEQPKFLGIREQRVTGLMIFVLMGCSVFMTAVLKFIPMPVLYGVFLYMGVSSLQGIQFFD     924
            ********************:***.*****::*****************.***:***:**

mSlc4a10    RIKLFWMPAKHQPDFIYLRHVPLRKVHLFTVIQMSCLGLLWIIKVSRAAIVFPMMVLALV     1017
rSlc4a8     RLKLFGMPAKHQPDFIYLRHVPLRKVHLFTLVQLTCLVLLWVIKASPAAIVFPMMVLALV     986
mSlc4a8     RLKLFGMPAKHQPDFIYLRHVPLRKVHLFTLVQLTCLVLLWVIKASPAAIVFPMMVLALV     984
            *.*** ************************..*..** ***.** * *************
```

Figure S1. Fragments of the multiple sequence alignment of Slc4a8 from *Mus musculus* (mSlc4a8), *Rattus norvegicus* (rSlc4a8) and Slc4a10 from *Mus musculus* (mSlc4a10). Sequences were retrieved from the UniProtKB database; accession codes are Q8JZR6 for mSlc4a8, F1LUB7 for r Slc4a8 and Q5DTL9 for mSlc4a10. Residues from the S1 site are highlighted in bold and those coordinating Na^+ (D800, T804, and T847) in rat Slc4a8 structure (PDB: 7rtm) are underlined. We defined the S1 site by residues within 8.0 Å from K^+ and CO_3^{2-} ions in the rSlc4a8 structure.

Supplementary Figure 2

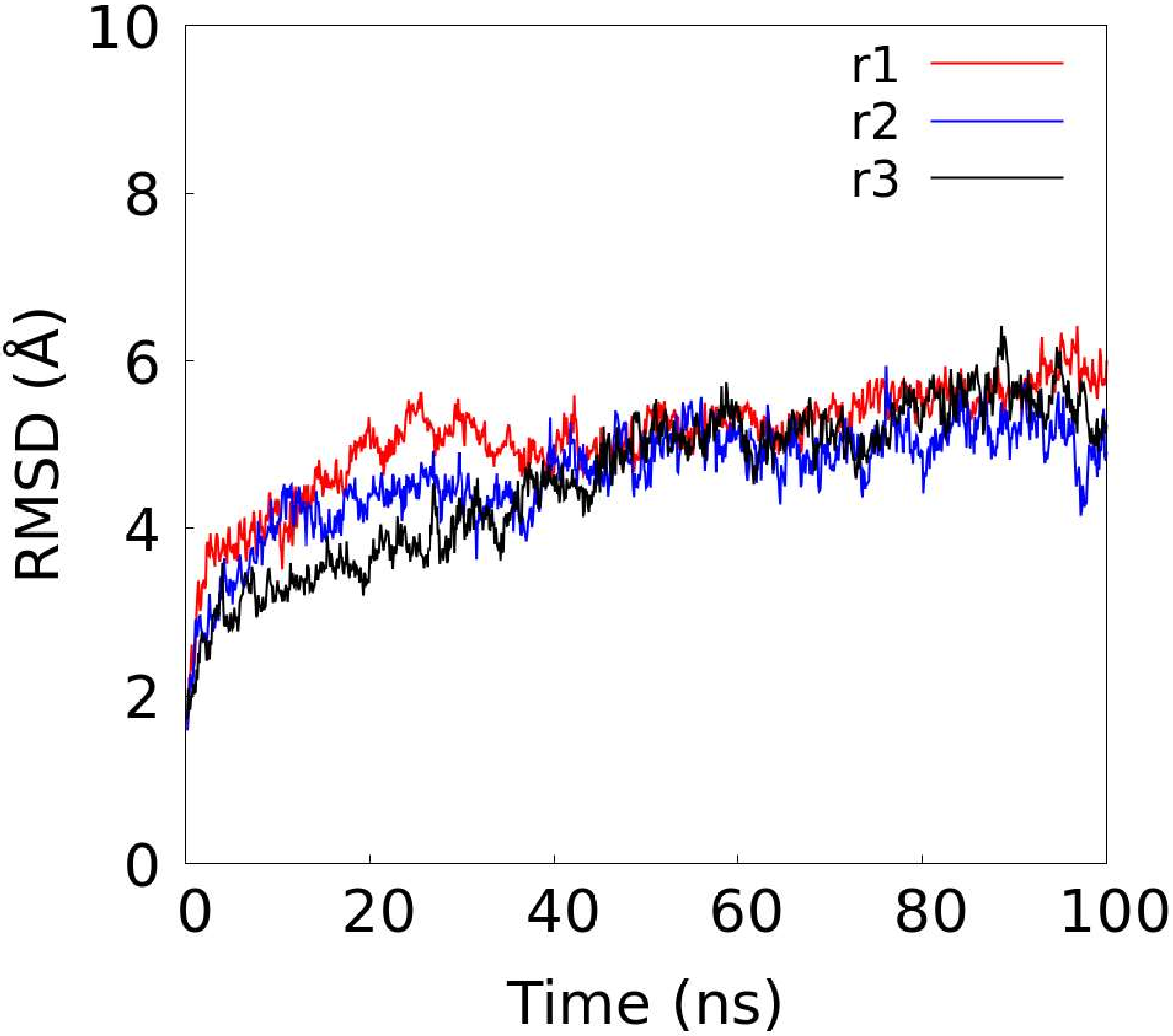

Figure S2. Root mean square deviation (RMSD) of backbone atoms during 100 ns for the three MD simulations (referred as r1, r2, and r3). RMSD was computed with reference to the first frame of each MD.
